# A GIS-based method for household recruitment in a prospective pesticide exposure study

**DOI:** 10.1186/1476-072X-7-18

**Published:** 2008-04-30

**Authors:** Justine LE Allpress, Ross J Curry, Carol L Hanchette, Michael J Phillips, Timothy C Wilcosky

**Affiliations:** 1 RTI International, 3040 Cornwallis Road, Research Triangle Park, North Carolina, USA; 2 Department of Geography & Geosciences, University of Louisville, Louisville, Kentucky, USA

## Abstract

**Background:**

Recent advances in GIS technology and remote sensing have provided new opportunities to collect ecologic data on agricultural pesticide exposure. Many pesticide studies have used historical or records-based data on crops and their associated pesticide applications to estimate exposure by measuring residential proximity to agricultural fields. Very few of these studies collected environmental and biological samples from study participants. One of the reasons for this is the cost of identifying participants who reside near study fields and analyzing samples obtained from them. In this paper, we present a cost-effective, GIS-based method for crop field selection and household recruitment in a prospective pesticide exposure study in a remote location. For the most part, our multi-phased approach was carried out in a research facility, but involved two brief episodes of fieldwork for ground truthing purposes. This method was developed for a larger study designed to examine the validity of indirect pesticide exposure estimates by comparing measured exposures in household dust, water and urine with records-based estimates that use crop location, residential proximity and pesticide application data. The study focused on the pesticide atrazine, a broadleaf herbicide used in corn production and one of the most widely-used pesticides in the U.S.

**Results:**

We successfully used a combination of remotely-sensed data, GIS-based methods and fieldwork to select study fields and recruit participants in Illinois, a state with high corn production and heavy atrazine use. Our several-step process consisted of the identification of potential study fields and residential areas using aerial photography; verification of crop patterns and land use via site visits; development of a GIS-based algorithm to define recruitment areas around crop fields; acquisition of geocoded household-level data within each recruitment area from a commercial vendor; and confirmation of final participant household locations via ground truthing. The use of these procedures resulted in a sufficient sample of participants from 14 recruitment areas in seven Illinois counties.

**Conclusion:**

One of the challenges in pesticide research is the identification and recruitment of study participants, which is time consuming and costly, especially when the study site is in a remote location. We have demonstrated how GIS-based processes can be used to recruit participants, increase efficiency and enhance accuracy. The method that we used ultimately made it possible to collect biological samples from a specific demographic group within strictly defined exposure areas, with little advance knowledge of the location or population.

## Background

### Pesticide exposures and health outcomes

The U.S. Environmental Protection Agency has defined pesticides as substances that are intended to prevent, destroy, repel or mitigate pests. Pests include insects, animals, weeds, fungi and microorganisms such as bacteria and viruses [1]. Although pesticides are associated primarily with the agricultural industry, many household products are also pesticides. These include flea and tick treatments, cockroach sprays and baits, disinfectants and sanitizers, and lawn and garden products.

Animal bioassays have shown many pesticides to be carcinogenic and some are known or suspected human carcinogens. Agricultural Health Study results have indicated that prostate and ovarian cancer incidence rates are elevated for male and female pesticide applicators, respectively [2]. Pesticides have been linked with cancers in children including leukemia, neuroblastoma, non-Hodgkins lymphoma, Wilm's tumor, Ewing's sarcoma, soft-tissue sarcoma, and cancers of the brain, colorectum and testes [3]. The link to cancer in children is of particular concern as young children are at greater risk from pesticide exposure than adults are, due to their higher skin to body mass ratio, hand-to-mouth activities, crawling and floor play that bring them into increased contact with soil and dust [4-6]. A high tendency to spend time indoors also increases possible exposure as residue is likely to persist longer indoors because sun, rain and soil microbial activity are not present to aid degradation [7]. With non-point-source pesticide contamination from agricultural applications occurring in water supplies, soil and airborne particulate matter [8-12] and only 10–15% of pesticides applied reaching target crops [8] it is important to develop accurate methods of measuring pesticide exposure. Recent advances in GIS technology and remote sensing (e.g. satellite images and aerial photography) have provided new opportunities to collect ecologic data on pesticide exposure.

### Geographic information systems, pesticide exposure studies and household recruitment

A number of studies have used GIS and remote sensing technologies to aid in agricultural pesticide exposure research. Many of these incorporated spatial functions such as distance measurement, buffering, and overlay analysis. Most used historical data on crops and their associated pesticide applications and measured residential proximity to agricultural fields.

Schreinemachers [13] used county-level data on wheat acreage to examine birth defects in the upper Midwest. Other studies used residential proximity to agricultural fields as a surrogate for exposure [14-16]. Area-based measures have been used to estimate crop acreages within specified buffer zones and, in some cases, model probabilities of pesticide use [17-20]. Data from California's pesticide use reporting (PUR) database have been used to examine a number of health outcomes, using a combination of distance- and area-based measures [21-26]. In some studies, pesticide use data has been linked to available land use maps [24,25]. Brody et al. have used GIS-based exposure modelling to examine historical patterns of pesticide use on Cape Cod [27,28].

Due to time and financial constraints, or an historical timeframe, very few of these studies collected environmental and/or biological samples from household members. The challenges faced by researchers in the analysis of household samples include: 1) identification and recruitment of study participants, 2) estimation of exposure and/or verification of pesticide use, and 3) accurate measurements of distance between study homes and pesticide application sites. Many pesticide study populations are drawn from disease registries or health outcomes databases, such as: state cancer registries [21,23,28], birth records or birth defects registries [14,17,20,25], Medicare databases [26], and population-based disease studies [18,19,24].

There are some exceptions, however. For their study of organophosphate metabolite concentration, Royster et al. [16] recruited children at local clinics where measles, mumps, rubella (MMR) shots were being administered. Koch et al. [29] contacted potential participants for their pesticide exposure study at Women, Infants and Children (WIC) clinics in central Washington State.

The identification of study fields and recruitment of study participants is a complex process that is often glossed over in reports of pesticide research. In this paper, we present a cost-effective, GIS-based method for crop field selection and household recruitment in a prospective pesticide exposure study. For the most part, our multi-phased approach was carried out in a research facility, but involved two brief episodes of fieldwork for ground truthing purposes. The method presented here is part of a larger study designed to examine the validity of indirect pesticide exposure estimates by comparing measured exposures in household dust, water and urine with records-based estimates that use crop location, residential proximity and pesticide application data. The timing of field identification, household recruitment and collection of biological samples was critical to our study, due to the half-life of atrazine, which is about 60 days in topsoil.

The use of GIS was central to the process of household recruitment. In the initial stages, we used it for the identification of potential study fields, the spatial definition of household recruitment areas, and mapping of study households. In later stages (not reported here), we used it for distance and directional measurements. The methods that we used to recruit household members ultimately made it possible to collect biological samples from a specific demographic group within strictly defined exposure areas, with little advance knowledge of the location or population.

## Methods

### Study area selection

Our study focused on the pesticide atrazine, a broadleaf herbicide used in corn production and one of the most widely-used pesticides in the U.S. We chose Illinois as our study state because of its high corn production and heavy atrazine use (Table 1). In 2002, the year our fieldwork commenced, Illinois ranked second among U.S. states in corn production, with 11 million acres planted. Atrazine was applied to 72% of the area planted in corn [30]. Our goal was to select study fields that met our specifications, initially using GIS and records-based methods to identify potential candidates, then validating or rejecting these choices during field visits. As part of our household sampling strategy, we would then use GIS buffering functions to select households at different distances from the field boundaries.

**Table 1:** Atrazine Use in Illinois, 1999–2003

**Year**	**% of Corn Acreage with Atrazine**	**Lbs/Acre**
1999	84	1.08
2000	81	1.09
2001	88	1.22
2002	72	1.20
2003	77	1.24

Source: Illinois Agricultural Statistics Service [30].

### Identification of potential study (candidate) fields

For inclusion in the study, a field had to be: 1) planted in corn during the spring of 2003, as biological and environmental samples would be collected after atrazine applications; 2) at least 1000 meters away from a "conflict field," i.e. any other field planted in corn that could also be treated with atrazine, thus potentially making determination of exposure source problematic, and 3) within 200 meters of a moderately populated residential area. Our study team used aerial photographs from the Illinois Natural Resources Geospatial Data Clearinghouse [31] to visually identify potential field locations. We downloaded and examined 284 Digital Orthophoto Quadrangles (DOQs), taken in 1998 and 1999, from 29 counties. Because aerial photographs are taken during the winter months, when leaves are off deciduous trees, it was not possible to identify specific crops. Fields were identified as large clearings with little or no vegetation.

Our preliminary review indicated that the best field candidates would be on the edge of medium to large towns, thus allowing for an appropriate housing density moving away from the field's edge. From these photographs, we identified 95 potential fields from 17 counties. Given the large number of cornfields in Illinois, one might ask why our examination included so many counties. The reason is that cornfields are so ubiquitous that it was very difficult to find "isolated" fields, i.e. fields far enough away from conflicting fields.

**Figure 1:**
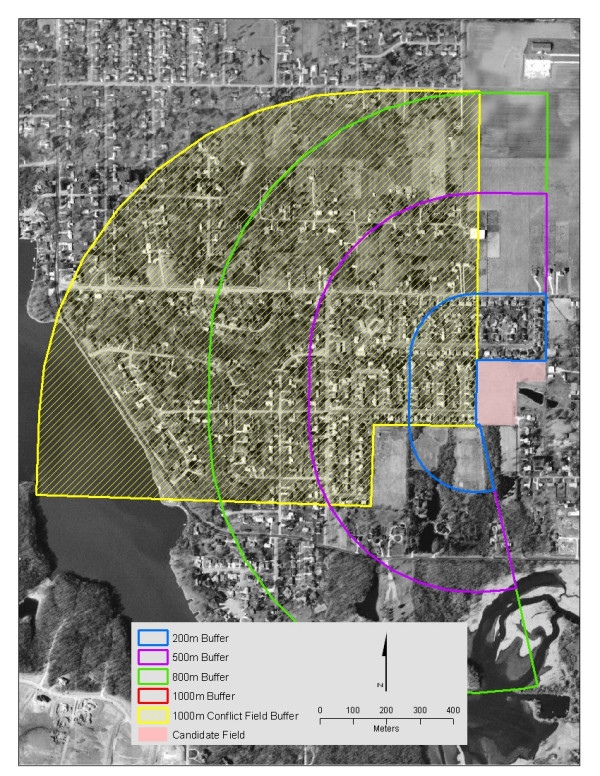
**Candidate field buffers and conflict field resolution**. The candidate field is located in the center of the aerial photo, with buffers of 200 (blue), 500 (purple), 800 (green) and 1000 (red) meters. Potential conflict fields are to the south, with a 1000-meter buffer displayed in yellow.

For each of the 95 potential fields, we digitized the field boundary closest to target households using Environmental Systems Research Institute's (ESRI) ArcGIS software. We then created buffers at distances of 200, 500, 800, and 1,000 meters from the edge of the field. These buffers were based upon studies of pesticide spray drift [e.g. [32]] and served two purposes: 1) they allowed us to identify potential conflict fields and 2) in accordance with our sampling strategy, residences could be categorized by distance from field boundaries, as other pesticide studies have done [17,18]. When potential conflict fields were identified, they were buffered by 1000 meters. Using these methods, we were able to predict how much of the study area might be lost to conflict fields. Figure 1 shows a potential candidate field with the four buffer zones. The area within 1000 m of the conflict fields is shown by the yellow line. In this particular example, if the candidate and conflict fields were all planted in corn during the sampling period (spring 2003), the candidate field would be rejected due to the fact that most of the residences were within 1000 m of a conflict field. If corn was planted in the candidate field, but not in either of the conflict fields, the field would be a valid candidate.

### Crop verification

Without ground truthing, there was no way to determine which crops were being grown in each field. Field selection was reliant on aerial photography taken in 1998 and 1999 because processed, up-to-date satellite imagery was not available for our large study area and our project budget did not include funds for image processing. Additionally, many of the areas identified for possible study were on the edges of towns experiencing population growth and new housing development. Because such construction could completely change the study area as it had been defined, or remove the selection of a field as a candidate altogether, it was impossible for the study team to predict which fields would actually be eligible for the study without traveling to Illinois to confirm crop patterns and current land use.

We conducted fieldwork in fall 2002 and spring 2003. The purpose of the 2002 visits was to verify crop and land use patterns and eliminate fields that were not candidates. This would provide us with adequate planning time to recruit study participants prior to the spring 2003 planting. Due to the fact that corn and soybeans are typically rotated annually as a "best management practice" for better crop yields [33,34], we sought to identify 2002 fields with soybean residue, assuming that they would be planted in corn the following spring. Hence, we used crop rotation practices as a predictor of field utilization for 2003.

**Figure 2:**
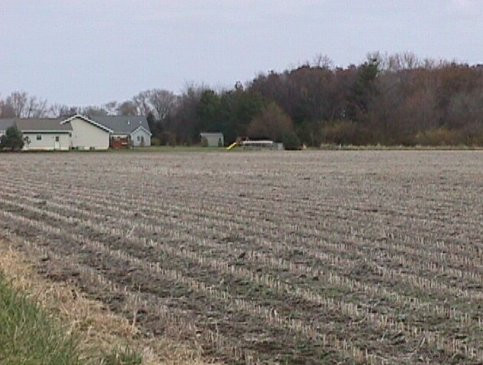
**Potential candidate field with soybean residue**. Field with soybean residue photographed in fall 2002.

In order to facilitate visits to all of the potential candidate fields we reduced the number of candidate fields to 80 and split them into two groups of 40 to enable separate field trips covering different areas of the state. We used paper maps of each field, similar to the version shown in Figure 1, and marked changes in a field's boundary and the local landscape. We photographed field boundaries in the direction of houses and noted potential barriers to pesticide drift, such as heavy vegetation. Most importantly, we documented crop information. We recorded the same set of information for all conflict fields. If we found soybean residue on the candidate field (Figure 2), we predicted that corn would be grown the following year. If we found corn residue on a conflict field we surmised that soybeans would likely be grown the following year, and the field would no longer pose as a conflict exposure for the candidate field.

At the end of the two initial field trips, 34 potential fields in 10 counties remained in the study. Those removed from consideration either had corn residue on the fields at the time of observation, suggesting that soybeans would be the crop for the following year, or the location was no longer a crop field.

### Spatial definition of recruitment areas

The next step was to identify households eligible for participant recruitment. Based on information from the fall 2002 site visits, boundaries for some fields were redrawn and new buffers created. Our goal was to develop an appropriate sample size of children living within each of the buffer zones (200, 500, 800, 1000 meters). This required the following information: 1) household composition – occupants' ages, type of dwelling (e.g. single family, apartment); 2) household location, i.e. latitude and longitude; and 3) contact information – address and phone number. After contacting marketing list vendors, we chose a vendor that provided information about all households fitting the study requirements that were within a specified radius from any known address that we provided. This method was determined to be the most cost-effective, as we could locate an address at the center of the study area and then create a radius to incorporate all the homes within the study buffers. Because fees were based on the number of addresses provided by the vendor, one of our challenges was to develop a method to capture all of the homes within the study areas while eliminating areas that did not meet our requirements, thus keeping costs down.

**Figure 3:**
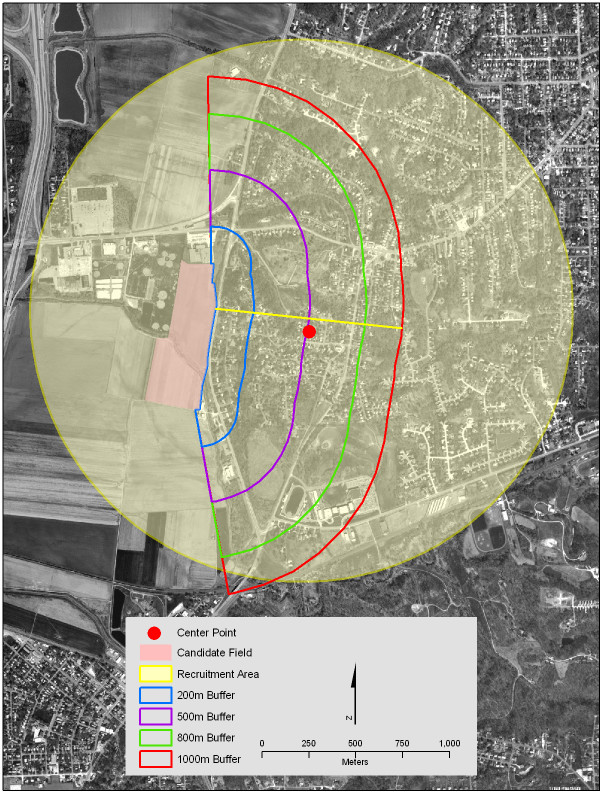
**Center point of study area and resulting recruitment boundary**. Using GIS functions, a straight line was drawn from the center of the field boundary arc closest to the residential area past the centroid of the 1000-meter buffer, to the edge of the 1000-meter buffer. The center point of this line was designated as the recruitment area centroid. The yellow line was created by buffering the address closest to the recruitment area centroid at a distance that included all homes in the recruitment area.

To achieve this, we developed an algorithm that we applied to all study fields. We located the centroid of the 1,000 m field buffer, as well as the half-way point along the field boundary (arc) closest to the study area. A straight line was drawn between these two points, running from the field boundary to the edge of the 1,000 m buffer. The center point of this line was designated as the center point of the study area (Figure 3).

Next, we had to find a real address as close as possible to this center point. This was a multi-step process. First we overlaid TIGER 2000 streets on the study area and identified the road closest to the center point. Using the address range along the road segment as a guide, we selected a fictitious address on the street and found a corresponding ZIP+4 for the address on the U.S. Postal Service web site [35]. When this was completed for all fields, we sent the ZIP+4 data to the vendor, who provided geocoded addresses for all homes in each ZIP+4. These addresses were mapped, enabling us to identify the address closest to the center point.

Finally, we determined the size of the radius needed to capture all potential candidate households in the study area by buffering the central address in increments of one-tenth of a mile (a requirement set by the vendor) until all of the homes in the study area were contained within the radius. On completion, we provided the central address and radius length to the data vendor.

### Mapping and evaluation of potential participants

The vendor provided geographic coordinates and other data about all homes in the specified study area. Using these data we identified all single-family homes with at least one child aged 4 or younger. For each study field, we created a GIS layer consisting of homes that met the study criteria and overlaid this with the aerial photography, buffer zones, and conflict fields. From this procedure we were able to determine the approximate number of potential candidate households in the area, as well as how these households were dispersed across the four buffer zones. In accordance with our study design, we determined that the optimum number of participating households in a study area was six, with two in each of the 0 to 200 m and 800 to 1000 m buffers, and one household in each of the other two buffers. From this initial overlay, it was possible to determine whether any of the fields were unacceptable due to a lack of potential household participants or because of inconsistently-spaced households throughout the study area. Some homes were also excluded because of their proximity to conflict fields. At the end of this process, 24 candidate fields remained, located between Rockford and East St. Louis, Illinois.

### Verification of house location and crop

In late spring 2003, we made two field trips to Illinois with the primary goal of verifying that corn was being grown in the candidate fields (and not in any conflict fields). We also needed to ascertain that the geocoded homes of potential recruitment households were located accurately. Mapping the residential location of study participants is an important component of pesticide exposure studies, as it allows researchers to measure distance and direction from agricultural fields. In most cases, address geocoding, which interpolates where an address falls along a street centerline, is used to assign geographic coordinates to residences. Much has been written about the accuracy of geocoding methods for epidemiologic studies [36-38]. When tax parcel databases are available, they can be used with a higher level of accuracy.

**Figure 4:**
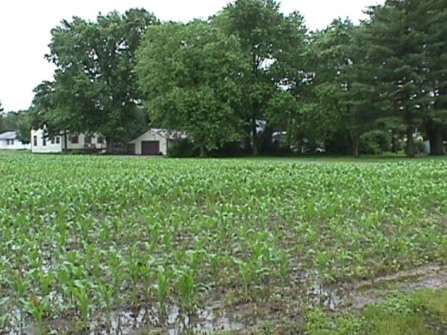
**Corn growing in candidate field**. Field confirmation of corn in study field, photographed in spring 2003.

Google Earth was launched in October 2004, too late for use in our study. For record-keeping and editing, we used ESRI's ArcPad software on a handheld computer with a GPS unit attached. Our application contained aerial photography and map layers of house locations, field boundaries, buffers, and conflict fields. Figure 4 shows a potential candidate field with corn growth visible. Once crop verification was achieved, we confirmed that the field boundaries were correct. If the field had been delineated properly, the next task was to confirm actual house locations.

Using address lists of all housesholds that met our study criteria, we navigated to each household and compared the geocoded point to the actual house location. Many of the geocoded points were inaccurate. To resolve this, we edited the shapefile using ArcPad and moved each point to its corresponding rooftop on the aerial photograph. Making edits in the field reduced the chance of inaccuracies during data transfer from paper to digital formats. In several cases, we discovered that the correct location of the house put it into a different buffer zone or even outside the study area. The inaccuracy of the original geocoded houses confirmed that the fieldwork verification process was integral to the accuracy of the study's final results. During this process we visited and verified the locations of 304 houses. At the conclusion of this stage of the investigation 21 fields remained viable for the study.

## Results

**Figure 5:**
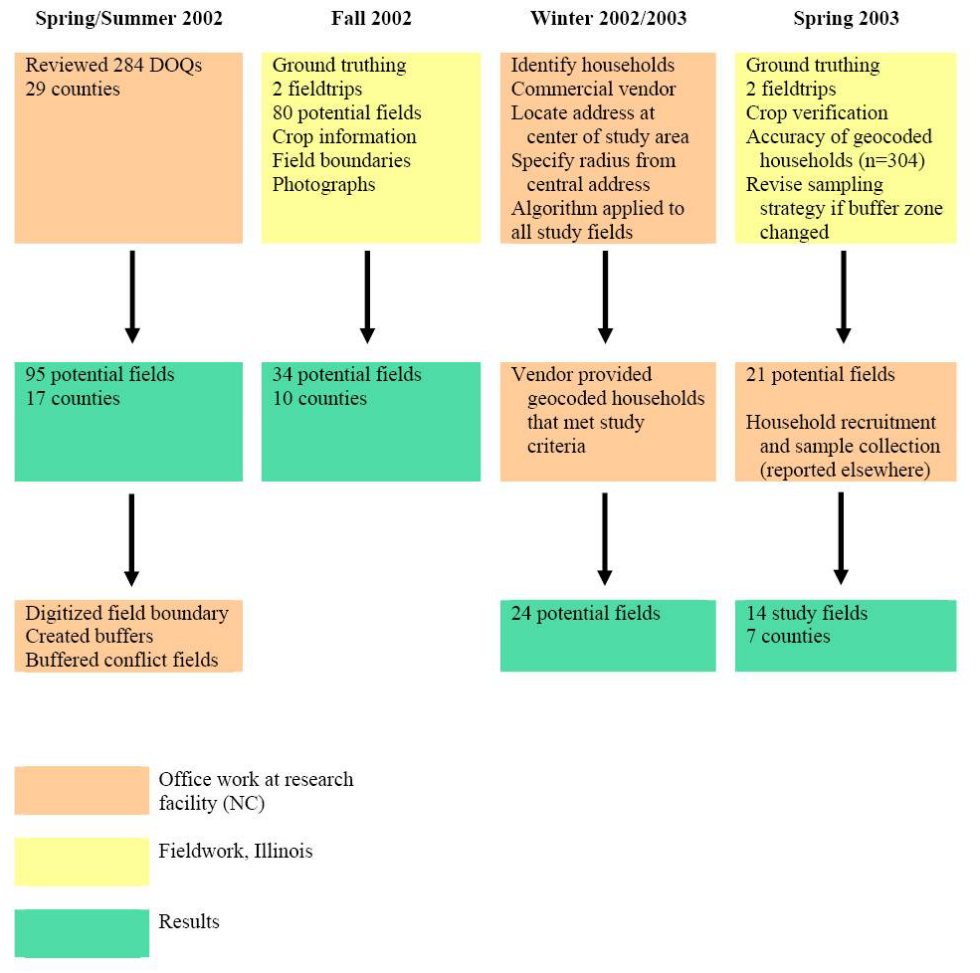
**Flowchart showing sequence of methods used for household recruitment**. Major tasks and timelines are diagrammed and color-coded by location and/or activity. Pink boxes indicate tasks that were carried out in the office, using GIS; tasks in yellow boxes were carried out during fieldwork in Illinois, and green boxes show number of candidate fields resulting from tasks performed during each major time period.

Figure 5 is a flowchart summarizing the sequence of the steps described in the Methods section, above. Using these methods, we were able to identify individual households that would be contacted for possible participation in the study. We achieved this by importing the house layers from ArcPad back into ArcGIS and displaying these layers over the buffers that had been updated with any changes observed from the field. After reviewing the verified household locations it was possible to finally see how the houses were spread through the buffer zones for each of the candidate fields. This made it possible to identify any fields that would not be feasible to study due to the dispersion of houses in the study area. We had decided that the optimum situation was one where all of the candidate households participating in the study were located in a relatively straight line moving away from the field border through the buffers. This was not possible for all fields, but we attempted to follow this guideline. The houses that most closely matched this pattern were identified and listed for future contact by recruiters.

**Figure 6:**
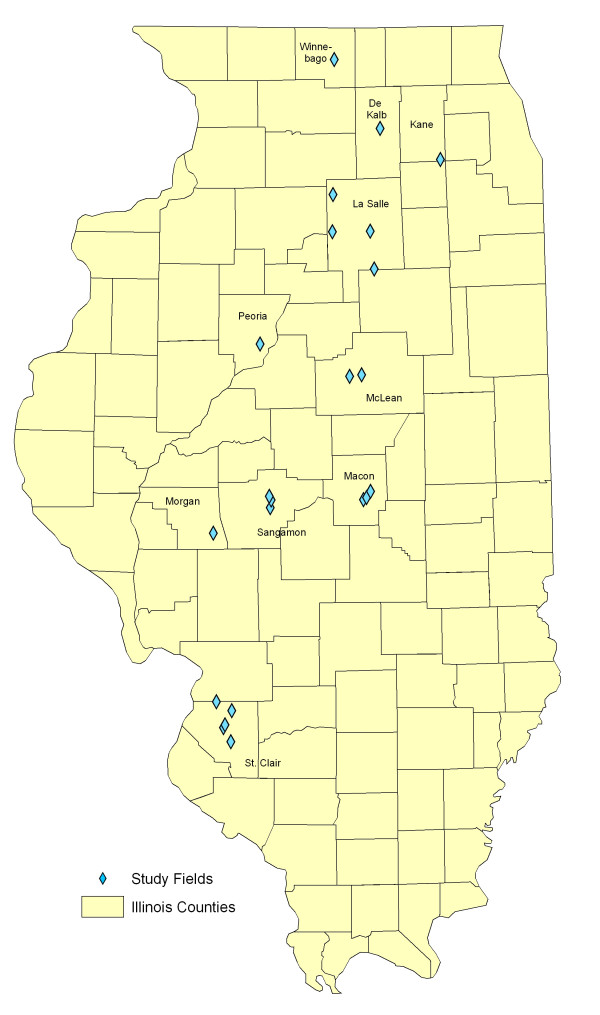
**Location of final study fields**. Map of Illinois counties showing the locations of the final fields included in the study. Field locations are represented by blue diamonds.

Household recruitment and sample collection procedures are being reported elsewhere, however, we ultimately enrolled 58 participants from 14 study fields in seven Illinois counties. Field locations are shown in Figure 6. Table 2 provides information about the study fields and shows the distribution of households across buffer areas.

**Table 2:** Study Fields and Household Distribution Across Buffer Areas

**Field ID**	**Field Size in Acres**	**Total Households**	**0–200 m**	**201–500 m**	**501–800 m**	**801–1000 m**
A	38	4	1	0	0	3
B	18	3	0	1	1	1
C	75	5	1	1	1	2
H	66	5	1	1	1	2
I	54	6	2	2	1	1
J	156	4	1	1	1	1
M	479	5	1	1	1	2
P	25	4	2	0	0	2
Q	17	5	1	0	2	2
R	6	4	2	1	0	1
S	23	4	2	0	1	1
U	29	2	1	0	0	1
V	139	3	1	0	1	1
W	75	4	1	1	1	1

## Discussion

In recent years, there has been a proliferation of epidemiologic research that utilizes GIS and spatial analysis. One of the greatest challenges of these studies is the accurate assessment of exposure. Too often, proximity is used as a surrogate for exposure, without reliable information to back up its use. The two main objectives of our larger research have been to 1) determine whether there is a clear exposure gradient with distance from corn fields and 2) evaluate the correspondence between predicted vs. actual exposures by comparing record-based methods with biological and environmental samples. The results of our larger study are being reported elsewhere, but the initial first steps of identifying study fields and participant recruitment areas have been reported here.

We successfully implemented several procedures designed to meet the requirements of locating candidate fields and homes. Through aerial photograph interpretation, we were able to identify potential fields and recruitment areas; however, ground truthing was necessary to confirm these selections. Without fieldwork, this portion of the investigation would have been impossible. Once in the field, the utilization of digital technologies with ArcPad aided in data collection and increased accuracy of field delineation and household location.

Using geocoded points provided by marketing list vendors was integral to the success of our task. However, geocoding as a means for precise address location is not infallible. Without ground truthing the geocoded results, we would have been unable to accurately locate homes that possessed the desired demographic characteristics in relation to candidate fields, which would have significantly compromised the project.

Fieldwork always has its challenges and, in this case, timing was critical since it relied on the planting and harvest cycles of crops. We did not anticipate the loss of so many potential candidate fields to surburban housing development. Despite these challenges, we were able to accurately and efficiently place homes within the study area and locate those that met the criteria imposed by the project. None of this would have been possible without the use of GIS and digital spatial technologies.

We have presented a novel approach to the complex process of selecting study fields and identifying households for the recruitment of study participants. However, in the past two-three years, new technologies and data products have become available that could have enhanced our multi-step process. In October 2004, Google Earth was launched. With up-to-date aerial photography, the use of Google Earth may have precluded the need to ground truth geocoded addresses.

In 1996, the U.S. Department of Agriculture (USDA) implemented the Cropland Data Layer (CDL) program [39]. Each year, this program produces raster, georeferenced, categorized land cover data layers for states that are major crop producers. The focus is on cropland and the purported accuracy rate for major crops ranges from 80% to the high 90s. These data layers were not available at the time of our study, but are useful for historical or landuse change analyses.

In order to evaluate the accuracy of Cropland Data Layer corn and soybean classifications for our study, we conducted a retrospective analysis using DOQs of 46 fields, on which we had identified and marked crop types during our 2002 and 2003 field visits. Of these 26 (57%) were classified correctly and 4 fields (9%) were covered by clouds. In general, accuracy was greater for larger fields. The two most common misclassifications were: 1) soy or corn was classified as pasture/grassland and 2) soy and corn pixels were interspersed. Given the rate of accuracy and the small size of some of our study fields, ground truthing would have been necessary to our study even if the satellite imagery data had been available in a more timely manner.

An important aspect of our study design was the premise of crop rotation as a means of predicting the following year's crop. Table 3 shows the acreage and percentage of soybean crop area that was rotated to corn from one year to the next, and vice versa, from 1999–2003. On average, the "annual" corn/soybean rotation occurred on about two-thirds of Illinois's corn/soy acreage. Among our final 14 study fields, the annual corn/soy rotation occurred in 50% (7) of the fields; however, the actual number is probably 86% (12), due to the misclassification patterns noted in the previous paragraph. Overall, the practice of corn/soybean rotation was less prevalent than we expected and is due to become even less common with the ethanol boom and the resulting increase in corn acreage [40].

**Table 3:** Corn/Soy Rotation, 1999–2003, Illinois

**SOY TO CORN**				
	**99–00**	**00–01**	**01–02**	**02–03**
**Acres**	6,912,864	6,919,889	6,858,970	6,750,093
**Percent**	67.37	68.98	67.24	63.73

**CORN TO SOY**				

	**99–00**	**00–01**	**01–02**	**02–03**
**Acres**	6,837,881	6.883,277	6.817,839	6,938,986
**Percent**	61.82	66.00	66.33	64.29

Source: USDA/NASS Cropland Data Layer, 1999–2003.

While the Cropland Data Layer focuses on cropland, it also contains information about other landuses, including urbanization. Between the years 1999–2003, Illinois lost an average of 4–5% of its corn/soy acreage annually to urbanization, much of it in the form of new housing. We certainly noted this trend during fieldwork and lost many potential study fields to it.

## Conclusion

We have demonstrated how GIS-based processes can be used to recruit participants, increase efficiency and enhance accuracy, resulting in the collection of biological samples from a specific demographic group within strictly defined exposure areas, with little advance knowledge of the location or population. In addition to being cost-effective, our iterative, multi-pronged method provided us with the quality control needed for our study. If we had not used this methodology, we would have comprised quality and accuracy. Inaccurate geocoding would have placed some potential study houses in the wrong buffer and invalidated our sampling strategy. Without field checks, time would have been wasted in geocoding homes in invalid study fields, such as those that were not planted in corn or had disappeared due to suburban development. While our methodology may require refinement, in light of new data and technologies, it provides a valuable framework for study field identification and household recruitment.

## Competing interests

The authors declare that they have no competing interests.

## Authors' contributions

All authors are responsible for the project design. JLEA and RJC carried out most of the GIS analysis. JLEA, RJC, CLH and MJP conducted the fieldwork. JLEA and CLH wrote the bulk of the manuscript, with contributions from the other authors. TCW was the project PI. All authors read and approved the final manuscript.
